# Rumen bacterial community responses to DPA, EPA and DHA in cattle and sheep: A comparative in vitro study

**DOI:** 10.1038/s41598-019-48294-y

**Published:** 2019-08-14

**Authors:** D. Carreño, P. G. Toral, E. Pinloche, A. Belenguer, D. R. Yáñez-Ruiz, G. Hervás, N. R. McEwan, C. J. Newbold, P. Frutos

**Affiliations:** 1 Instituto de Ganadería de Montaña (CSIC-Universidad de León), Finca Marzanas s/n, 24346 Grulleros, León, Spain; 2 Institute of Biological, Environmental and Rural Sciences (IBERS), Animal and Microbial Sciences, Aberystwyth University, Aberystwyth, Ceredigion, SY23 3EB United Kingdom; 3 Estación Experimental del Zaidín (CSIC), Profesor Albareda 1, 18008 Granada, Spain; 4 School of Pharmacy and Life Sciences, Robert Gordon University, Aberdeen, AB10 7GJ United Kingdom; 5 Scotland’s Rural College (SRUC), Kings Buildings, Edinburgh, EH9 3JG United Kingdom

**Keywords:** Next-generation sequencing, Bacteria, Microbiome

## Abstract

The role of marine lipids as modulators of ruminal biohydrogenation of dietary unsaturated fatty acids may be explained by the effects of their n-3 polyunsaturated fatty acids (PUFA) on the bacterial community. However, the impact of individual PUFA has barely been examined, and it is uncertain which bacteria are truly involved in biohydrogenation. In addition, despite interspecies differences in rumen bacterial composition, we are not aware of any direct comparison of bovine and ovine responses to dietary PUFA. Therefore, rumen fluid from cannulated cattle and sheep were used as inocula to examine *in vitro* the effect of 20:5n-3 (EPA), 22:5n-3 (DPA), and 22:6n-3 (DHA) on the bacterial community. Amplicon 16 S rRNA sequencing suggested that EPA and DHA had a greater contribution to the action of marine lipids than DPA both in cattle and sheep. Certain effects were exclusive to each ruminant species, which underlines the complexity of rumen microbial responses to dietary fatty acids. Based on changes in bacterial abundance, *Barnesiella*, *Prevotella, Paraprevotella, Hallela, Anaerovorax, Succiniclasticum, Ruminococcus* and *Ruminobacter* may be involved in the ruminal response in biohydrogenation to the addition of marine lipids, but further research is necessary to confirm their actual role in ruminal lipid metabolism.

## Introduction

Addition of marine lipids to the diet of ruminants has proven useful to increase the concentration of some potentially health-promoting fatty acids (FA) in milk and meat (e.g., *cis*-9 *trans*-11 conjugated linoleic acid)^1^, an effect which is mostly explained by rumen microbial activity^2,3^.

Marine lipids are rich in very long-chain n-3 polyunsaturated fatty acids (PUFA) that modulate the pathways of FA biohydrogenation (BH)^2,4^. Eicosapentaenoic acid (EPA, 20:5n-3) and docosahexaenoic acid (DHA, 22:6n-3) are mainly responsible for this action through their effect on the rumen bacterial community^4,5^. Some *in vitro* studies have suggested that the modulatory effects of EPA and DHA may differ^5,6^, which could be associated with differences in their toxicity towards certain rumen bacteria^7^. In addition, Toral *et al*.^6^ showed that docosapentaenoic acid (DPA, 22:5n-3), a less abundant n-3 PUFA in marine lipids, had a lower impact on *in vitro* C18 BH than EPA or DHA, but no report has compared their effects on the rumen bacterial community.

The microbiology of ruminal FA metabolism remains poorly understood^4,8,9^ and next generation sequencing (NGS) platforms have rarely been used in this field^10–12^. Furthermore, most experiments on the effects of PUFA supplementation on the ruminal ecosystem have been conducted in cattle^8,12,13^, and few of them have focused on sheep^9,14^. Nevertheless, inherent differences in their rumen bacterial community^15,16^ may also imply differences in biohydrogenating bacteria, which could account for the specific response of bovine and ovine microbiota to EPA, DPA and DHA observed in a recent *in vitro* assay^6^. However, we are not aware of any comparative study on the effects of these n-3 PUFA on the rumen bacteria of these ruminant species.

On this basis, we established two hypotheses: 1) the major n-3 PUFA in marine lipids have different effects on the rumen bacterial structure, with DPA having a lower influence than EPA and DHA, and 2) interspecies differences in the ruminal microbiota of cows and sheep would be associated with distinct responses in the bacterial community, particularly biohydrogenating populations, to lipid supplementation. To test these hypotheses, 16S rRNA amplicon sequencing was used in an *in vitro* study to examine the effect of DPA, EPA and DHA on the rumen bacterial community of cattle and sheep.

## Materials and Methods

All protocols involving animals were approved by the Research Ethics Committee of the *Instituto de Ganadería de Montaña*, the Spanish National Research Council (CSIC) and the *Junta de Castilla y León* (Spain), following proceedings described in Spanish and EU legislations (Royal Decree 53/2013 and Council Directive 2010/63/EU).

### Batch cultures of rumen microorganisms

This assay is part of a larger study conducted to characterize the ruminal responses of cattle and sheep to major n-3 PUFA in marine lipids. The experimental design and methodology were extensively described in a first article^6^ that compared the effects of the PUFA on ruminal fermentation and digesta FA profile.

Briefly, the trial followed a 2 × 4 factorial arrangement with 2 ruminant species (bovine and ovine) and 4 PUFA treatments (DPA, EPA, DHA, and a control without additional FA). Batch cultures of rumen microorganisms were performed in Hungate tubes, using rumen inocula collected from 2 cannulated cattle and 2 cannulated sheep, and were repeated on 3 different days (replicates). All animals were fed the same high-concentrate total mixed ration, which was offered at estimated maintenance energy requirements^17^ to work under similar conditions in both species. Rumen fluid was obtained before feeding and strained through a nylon membrane (400 µm pore size). For each animal species, equal volumes of the 2 strained rumen fluids were combined and mixed (1:4) with phosphate-bicarbonate buffer^18^. Each incubation tube contained 12 mL of buffered rumen fluid and 120 mg of the total mixed ration fed to the animals, which provided, per kg of dry matter, 187 g of crude protein, 311 g of neutral detergent fiber and 18 g of total FA. The 3 n-3 PUFA [10-2205-9 (DPA), 10-2005-9 (EPA) or 10-2206-9 (DHA); Larodan, Solna, Sweden] were added at a dose of 2% of substrate dry matter (1 mg of PUFA/mL of rumen fluid), dissolved in ethanol 96% at 0.5% of the incubation volume and just before the buffered rumen fluid was dosed. Vials were then incubated under anaerobic conditions at 39.5 °C and gas accumulation was prevented through the insertion of a hypodermic needle in the rubber stopper. The reaction was stopped after 24 h by placing the tubes into ice-water for approximately 5 min. Samples were freeze-dried and stored at −80 °C until DNA extraction.

### DNA extraction

Freeze-dried ruminal digesta samples were thoroughly homogenised by stirring with a sterile spatula before DNA extraction, which was conducted using the Qiagen QIAmp DNA Stool Mini Kit (Qiagen Inc., Valencia, CA, USA), with the modification of a greater temperature (95 °C for 5 minutes) to improve cell lysis. The extraction was repeated twice for each sample, and these duplicates were combined and used as templates for NGS analysis. The DNA concentration and purity were measured by spectrophotometry (NanoDrop ND-100 Spectrophotometer; NanoDrop Technologies, Wilmington, DE, USA).

### Ion Torrent NGS analysis

Ruminal bacterial community was studied by NGS using an Ion Torrent Personal Genome Machine (PGM) system (Thermo Fisher Scientific, Leicestershire, UK)^19^. First, amplification of the V1–V2 hypervariable region of the 16S rRNA was carried out using the bacterial primers 27F and 357R^20^. The forward primer (AGAGTTTGATCMTGGCTCAG) carried the Ion Torrent Primer A-key adaptor sequence (CCATCTCATCCCTGCGTGTCTCCGACTCAG) and the reverse primer (CTGCTGCCTYCCGTA) carried the Ion Torrent Primer P1-key adaptor sequence A (CCTCTCTATGGGCAGTCGGTGAT) followed by a 10 nucleotide sample specific barcode sequence. The PCR was conducted in duplicate; a 25 μL reaction was prepared containing 1 μL of the DNA template (100 ng/μL), 1 μL of the forward primer (0.2 µM), 0.2 μL of the reverse primer (0.2 µM), 5 μL of the buffer with oligonucleotides (PCR Biosystems Ltd., London, UK), and 0.25 μL of bio HiFi polymerase (PCR Biosystems Ltd.). Amplification conditions were 95 °C for 1 min, then 22 cycles of 95 °C for 15 s, 55 °C for 15 s, 72 °C for 30 s and a final extension at 72 °C for 7 min. Resultant amplicons were visualised on a 1% (w/v) agarose gel to assess the quality of amplification before pooling the duplicate reactions.

The pooled PCR products were purified using Agencout AMpure XP beads (Beckman Coulter Inc., Fullerton, CA, USA), and DNA concentration was assessed using an Epoch Microplate Spectrophotometer (BioTek, Potton, UK) to enable equimolar pooling of samples with unique barcodes. Libraries were further purified using the E-Gel System with 2% agarose gel (Life Technologies Ltd, Paisley, UK). Purified libraries were assessed for quality and quantified on an Agilent 2100 Bioanalyzer with a High Sensitivity DNA chip (Agilent Technologies Ltd., Stockport, UK). The emulsion PCR was performed using the Ion Chef system with the Ion PGM IC 200 Kit (Thermo Fisher Scientific), and the sequencing with the Ion Torrent PGM (Thermo Fisher Scientific) system on an Ion PGM Sequencing 316 Chip v2 (Life Technologies Ltd).

Following sequencing, data were processed as described by de la Fuente *et al*.^19^. Briefly, sequences were transformed to FASTA format and sample identification numbers were assigned to multiplexed reads using the MOTHUR software environment (https://www.mothur.org/). Data were de-noised by removing low-quality sequences, sequencing errors and chimeras. The applied quality parameters were: maximum of 10 homopolymers, Q15 average over a 30 bp window, and no mismatches allowed with barcode and one maximum with primer. Chimera check was conducted using Uchime in both de novo and database driven modes. Then, sequences were cluster into operational taxonomic units (OTU) at 97% identity using CD-HIT-OTU pipeline (http://weizhong-lab.ucsd.edu/cd-hit-otu/). The number of reads per sample was normalised to the sample with the lowest number of sequences with Daisychopper (www.genomics.ceh.ac.uk/GeneSwytch/) followed by singleton read filtering^21^. Bacterial taxonomic information on 16S rRNA sequences was obtained by comparing against the Ribosomal Database Project-II. Raw sequences reads were deposited at the EBI Short Read Archive of the European Nucleotide Archive under accession number ERP104653.

### Statistical analysis

Before statistical analysis and because some data of OTU relative abundances did not satisfy the assumptions of normality, values were log-transformed.

The R-project software (www.r-project.org, version 3.2.2; “agricolae” and “vegan” packages) and relative abundances of each OTU were used to build dendrograms, with the complete-linkage method based on Bray-Curtis distances, and to create principal coordinate analysis (PCoA) plots for each ruminant species separately. The same software and data were employed to conduct a multivariate analysis of variance (MANOVA). The statistical model included the fixed effect of ruminant species (Sp; bovine and ovine), the PUFA treatment (control, DPA, EPA and DHA) and their interaction. For each species, pairwise comparisons were also conducted to elucidate differences between treatments, and adjusted for multiple comparisons using Benjamini and Hochberg’s method.

Observed species (i.e., number of distinct OTU), diversity indices (Chao1, Shannon and Simpson^22^) and the relative abundance of each OTU were analysed by ANOVA using the MIXED procedure of the SAS software package (version 9.4, SAS Institute Inc., Cary, NC, USA). The statistical model included the fixed effect of ruminant species, the PUFA treatment and their interaction. The incubation run and the inoculum nested within the species were designated as random effects. Means were separated through the pairwise differences (“pdiff”) option of the least squares means (“lsmeans”) statement of the MIXED procedure, and adjusted for multiple comparisons using Bonferroni’s method. The CORR procedure of SAS was used to generate Pearson correlation coefficients (*r*) between OTU relative abundances and ruminal C18 FA concentrations and fermentation parameters reported in the companion paper^6^.

Differences were declared significant at *P* < 0.05 and a trend toward significance at 0.05 ≤ *P* < 0.10. Least square means are reported.

## Results and Discussion

The sequencing of the bacterial 16 S rRNA amplicon generated an average of 66.922 (s.d. = 14.144) sequences per sample. After quality control, normalization and singleton read filtering, a total of 38,319 (s.d. = 77.6) sequences per sample were kept, which allowed identifying up to 2,199 OTU. A similar number of sequences was found in previous analysis of the rumen bacterial community by Ion Torrent NGS^19,23^. Given that the bacteria playing a dominant role in BH may be uncultured^8,14^, the use of NGS for determining populations affected by PUFA, including minor groups, would be particularly interesting. Nevertheless, changes in bacterial populations to PUFA addition may not be necessarily linked to an involvement in ruminal BH but to distinct sensitivities to the toxic effect of these fatty acids. Furthermore, modulation of enzymatic activity might play a role besides bacterial abundance^24^. Therefore, co-occurrence patterns of variation between rumen bacteria and rumen digesta FA profiles reported in Toral *et al*.^6^, which may help identifying candidate C18 biohydrogenating bacteria, will be discussed with much caution.

The hierarchical clustering analysis (Supplementary Fig. S1) showed the expected separation between ruminant species, which was also confirmed by MANOVA (*P* < 0.001; Supplementary Table S1) and agrees with previous comparative analysis of the rumen bacterial structure in cows and small ruminants^16,25^. A relatively better separation between EPA + DHA and control + DPA in sheep was observed in the hierarchical clustering analysis (Supplementary Fig. S1), but the interaction Sp × PUFA was not significant in the MANOVA analysis (*P* = 0.56).

**Figure 1:**
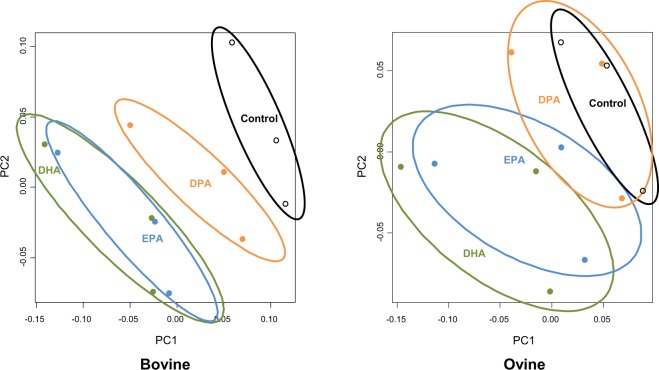
Principal coordinate analysis plot of bacterial 16S rRNA gene sequences after 24 h of *in vitro* incubation with rumen inocula of cattle and sheep. The incubated substrate was a total mixed ration containing no additional PUFA (control; black) or supplemented with 2% dry matter of docosapentaenoic acid (DPA; orange), eicosapentaenoic acid (EPA; blue), or docosahexaenoic acid (DHA; green). PC1 and PC2 = principal components 1 and 2, respectively.

Discrimination by PUFA treatment was clearer in the PCoA plot (Fig. 1). Consistent with the results of this latter method, the pairwise analysis separated EPA and DHA from the control in both species (*P* < 0.05), with no differences being detected between both FA (*P* > 0.33; Supplementary Table S1). In bovine cultures, DPA had a distinct impact on the structure of the bacterial community compared with that of EPA or DHA (*P* < 0.05), which seems to be supported by PCoA results. In ovine, however, the pairwise analysis confirmed the differences between DPA and EPA or DHA (*P* < 0.05), but not compared with the control (*P* = 0.23; Supplementary Table S1). This different response between cattle and sheep could be speculated to reflect the sensitivity of their bacterial communities to supplemental lipids. This might, in turn, contribute to explain some interspecies variations in the effects of PUFA on C18 BH^6^, such as the stronger inhibition of *trans*-11 18:1 saturation to 18:0 in bovine than in ovine cultures. Although we are not aware of published studies comparing the *in vivo* response of cattle and sheep to PUFA-rich supplements, interspecies differences to fish oil consumption were described in the rumen bacteria of cows and goats^25^.

**Figure 2:**
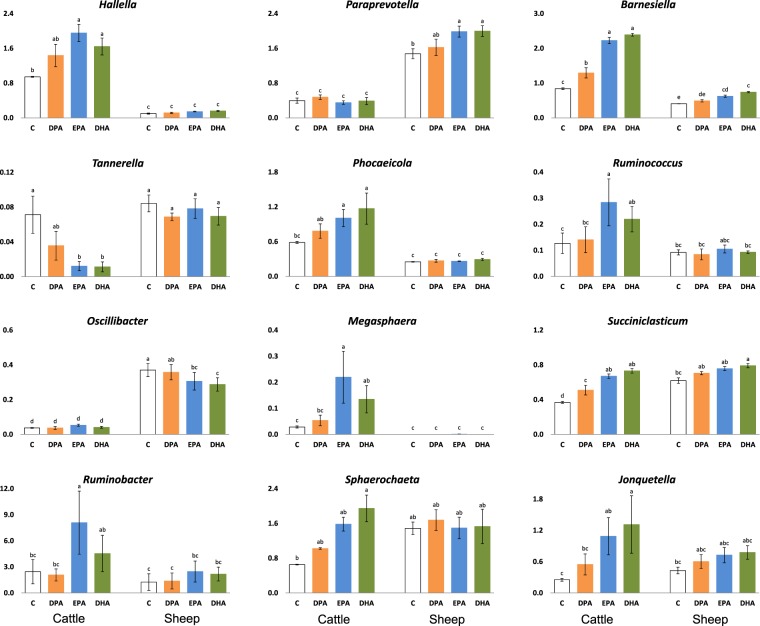
Relative abundances (% of total sequences, non-transformed values) of relevant bacterial genera (for which the effect of the interaction ruminant species × PUFA treatment was significant at *P* < 0.05; see Table 1) after 24 h of *in vitro* incubation with rumen inocula of cattle and sheep. The incubated substrate was a total mixed ration containing no additional PUFA (control, C; black) or supplemented with 2% dry matter of docosapentaenoic acid (DPA; orange), eicosapentaenoic acid (EPA; blue), or docosahexaenoic acid (DHA; green). Error bars represent the standard error of the mean. Within a genus, different superscripts (^a–d^) indicate significant differences due to the effect of the interaction ruminant species × PUFA treatment (*P* < 0.05), according to the statistical analysis reported in Table 1 and conducted with log-transformed values.

Table 1 reports the main effects of ruminant species and PUFA treatment on diversity indices and relative abundances of specific OTU, focusing on the most abundant bacterial groups and those affected by PUFA or previously suggested to be somehow related to rumen BH of C18 FA^8,11,26^. Results are expressed as log_10_ (n + c), and for reasons of clarity, the corresponding non-transformed means are also presented in Supplementary Table S2. In addition, the effects of PUFA are shown separately for each ruminant species in Fig. 2 (relevant genera for which the interaction Sp × PUFA was significant) and in Supplementary Fig. S2 (bacterial phyla).

**Table 1:** Diversity indices and relative frequencies (log-transformed data^a^) of bacterial 16S rRNA gene sequences of relevant phyla, families and genera after 24 h of *in vitro* incubation with rumen inocula of cattle and sheep^b^. Non-transformed means are reported in Supplementary Table S2.

	Cattle	Sheep	PUFA treatment				SED^c^	*P*-value^d^		
			Control	DPA	EPA	DHA		Sp	PUFA	Sp × PUFA
Diversity indices										
Observed species^e^	1220	1471	1377	1366	1332	1308	62.3	<0.001	0.413	0.728
Chao1	1620	1844	1732	1837	1737	1622	249.7	0.091	0.690	0.628
Shannon	5.23	5.20	5.30^ab^	5.32^a^	5.20^ab^	5.03^b^	0.133	0.590	0.030	0.083
Simpson	0.983	0.961	0.978	0.980	0.972	0.958	0.0083	<0.001	0.009	0.009
Taxonomic identification										
*Bacteroidetes*	2.03	2.05	2.04	2.04	2.02	2.04	0.006	<0.001	0.001	<0.001
Unclassified	1.85	1.87	1.86	1.87	1.85	1.88	0.013	0.017	<0.001	<0.001
*Prevotellaceae*	1.44	1.42	1.48^a^	1.45^ab^	1.41^bc^	1.39^c^	0.022	0.236	<0.001	0.102
Unclassified	1.00	0.96	1.00	0.99	0.97	0.95	0.019	<0.001	0.008	0.013
*Prevotella*	1.14	1.13	1.24^a^	1.16^b^	1.09^c^	1.06^c^	0.033	0.674	<0.001	0.184
*Hallella*	0.36	−0.03	0.10	0.16	0.21	0.19	0.034	<0.001	0.002	0.017
*Paraprevotella*	0.17	0.45	0.03	0.31	0.32	0.33	0.028	<0.001	0.061	0.005
*Porphyromonadaceae*	0.75	0.95	0.79	0.82	0.89	0.90	0.014	<0.001	<0.001	0.047
Unclassified	0.45	0.85	0.62	0.63	0.67	0.67	0.015	<0.001	<0.001	0.048
*Barnesiella*	0.44	0.23	0.24	0.30	0.38	0.41	0.001	<0.001	<0.001	<0.001
*Tannerella*	−1.09	−0.89	−0.89	−0.99	−1.03	−1.05	0.064	0.060	0.001	0.007
*Bacteroidales inc. sed*.	0.16	−0.07	−0.01	0.03	0.06	0.09	0.042	0.003	0.002	0.008
*Phocaeicola*	0.16	−0.07	−0.01	0.03	0.06	0.09	0.042	0.003	0.002	0.008
*Firmicutes*	1.58	1.54	1.56	1.55	1.57	1.56	0.012	0.012	0.130	0.613
Unclassified	1.19	1.20	1.18^c^	1.19^bc^	1.21^a^	1.21^ab^	0.013	0.494	<0.001	0.959
*Ruminococcaceae*	1.08	0.98	1.05^a^	1.03^ab^	1.04^ab^	1.02^b^	0.025	0.013	0.004	0.129
Unclassified	1.05	0.93	1.00^a^	0.98^ab^	0.99^ab^	0.97^b^	0.028	0.012	0.004	0.069
*Ruminococcus*	−0.49	−0.63	−0.60	−0.60	−0.50	−0.54	0.056	0.117	<0.001	0.004
*Oscillibacter*	−0.64	−0.29	−0.45	−0.46	0.47	−0.49	0.034	0.008	0.030	0.001
*Lachnospiraceae*	0.81	0.68	0.78	0.74	0.74	0.73	0.024	<0.001	0.063	0.749
Unclassified	0.51	0.46	0.05	0.49	0.49	0.47	0.029	0.037	0.827	0.052
*Butyrivibrio*	0.32	0.04	0.24^a^	0.16^b^	0.17^b^	0.16^b^	0.036	<0.001	0.010	0.614
*Pseudobutyrivibrio*	−0.17	−0.20	−0.14	−0.21	−0.19	−0.21	0.052	0.471	0.066	0.544
*Roseburia*	−1.34	−1.62	−1.55	−1.51	−1.41	−1.47	0.062	0.030	0.008	0.006
*Veillonellaceae*	−0.11	−0.01	−0.11	−0.06	−0.02	−0.06	0.287	0.040	<0.001	<0.001
Unclassified	−0.48	−0.11	−0.32	−0.27	−0.28	−0.30	0.040	0.010	0.010	0.014
*Megasphaera*	−0.86	−1.26	−1.17	−1.12	−0.94	−1.01	0.115	0.051	0.005	0.005
*Selenomonas*	−1.11	−1.43	−1.30	−1.28	−1.23	−1.26	0.056	0.001	0.237	0.237
*Anaerovibrio*	−0.89	−1.19	−1.08	−1.04	−1.00	−1.03	0.031	0.004	0.017	0.045
*Acidaminococcaceae*	0.08	0.14	0.06	0.10	0.13	0.15	0.014	<0.001	<0.001	0.004
*Succiniclasticum*	0.08	0.14	0.06	0.10	0.13	0.15	0.014	<0.001	<0.001	0.005
*Clostridiales inc. sed. XIII*	0.06	−0.03	−0.04^c^	0.00^b^	0.06^a^	0.04^ab^	0.026	<0.001	<0.001	0.121
*Anaerovorax*	−0.09	−0.20	−0.19^c^	−0.16^bc^	−0.10^a^	−0.12^ab^	0.029	0.004	0.003	0.901
*Proteobacteria*	1.24	1.06	1.11	1.11	1.12	1.16	0.233	0.005	<0.001	<0.001
Unclassified	0.93	0.71	0.81	0.81	0.84	0.81	0.031	0.015	0.075	<0.001
*Succinivibrionaceae*	0.88	0.75	0.74	0.75	0.93	0.85	0.043	0.049	<0.001	0.011
*Succinivibrio*	−0.25	−0.62	−0.51	−0.47	−0.35	−0.41	0.663	0.002	0.003	0.040
*Ruminobacter*	0.82	0.67	0.67	0.69	0.86	0.78	0.050	0.052	<0.001	0.018
*Tenericutes*^f^	0.97	1.00	1.10^a^	1.00^ab^	0.96^bc^	0.86^c^	0.058	0.547	<0.001	0.380
*Anaeroplasma*	0.97	0.99	1.10^a^	1.00^ab^	0.96^bc^	0.86^c^	0.058	0.566	<0.001	0.378
*Fibrobacteres*^g^	0.60	0.63	0.51^c^	0.59^b^	0.67^a^	0.70^a^	0.045	0.546	<0.001	0.541
*Spirochaetes*	0.61	0.64	0.59	0.61	0.63	0.65	0.037	0.531	0.041	0.015
*Spirochaetaceae*	0.59	0.60	0.56	0.59	0.61	0.63	0.041	0.784	0.027	0.016
*Sphaerochaeta*	0.43	0.47	0.39	0.44	0.47	0.49	0.047	0.308	0.007	0.003
*Synergistetes*^h^	0.16	0.14	0.04	0.12	0.21	0.24	0.045	0.492	<0.001	0.035
*Jonquetella*	0.15	0.13	0.02	0.10	0.20	0.23	0.048	0.519	<0.001	0.031
Other phyla^i^	0.71	0.53	0.64^a^	0.63^ab^	0.59^b^	0.62^ab^	0.033	0.002	0.008	0.059

^a–c^Within a row, different superscripts indicate significant differences (*P* < 0.05) due to the effect of PUFA treatment.

^a^Values were transformed to log_10_ (n + c), c being a constant of the same order of magnitude as the variable.

^b^The incubated substrate was a total mixed ration containing no additional PUFA (control) or supplemented with 2% dry matter of docosapentaenoic acid (DPA), eicosapentaenoic acid (EPA), or docosahexaenoic acid (DHA).

^c^Standard error of the difference.

^d^Probability of significant effects due to ruminant species (Sp), PUFA treatment, and their interaction (Sp × PUFA). In the pairwise analysis, *P*-values were adjusted for multiple comparisons using Bonferroni’s method.

^e^Number of distinct OTU.

^f^The *Anaeroplasmataceae* family comprises >99% of sequences within this phylum.

^g^The *Fibrobacter* genus comprises >99% of sequences within this phylum.

^h^The *Synergistaceae* family comprises >99% of sequences within this phylum.

^i^Sum of SR1, *Elusimicrobia*, *Lentisphaerae*, *Candidatus Saccharibacteria*, *Chloroflexi*, *Actinobacteria*, *Cyanobacteria/Chloroplast*, *Verrucomicrobia*, *Armatimonadetes* and *Fusobacteria*.

In relation to bacterial diversity (Table 1), the number of observed species (i.e., number of distinct OTU) and the Chao1 index were not significantly affected by PUFA treatment (*P* > 0.10), whereas the Simpson index showed a significant interaction Sp × PUFA (*P* < 0.01), the decrease with DHA being more marked in sheep (data not shown). In both ruminant species, the latter PUFA also reduced the Shannon index compared to DPA (*P* < 0.05), which had a weak effect on diversity indices. Overall, the relatively limited variations in diversity indices with PUFA treatments seem consistent with results from earlier *in vitro* and *in vivo* studies examining the effects of dietary marine lipid supplementation^11,14,27^.

As reported in Supplementary Fig. S2, the composition of the bacterial community showed the usual distribution pattern^19,23,28^, with most sequences being assigned to the *Bacteroidetes* phylum (47–60%), followed by *Firmicutes* (16–21%) and *Proteobacteria* (4–16%). The *Tenericutes* phylum (2–8%) was relatively abundant compared with *in vivo* studies (around 2% of sequences^11,23^), suggesting that *in vitro* conditions would favour its growth. This is supported by the lower *Tenericutes* abundance in the initial inocula (on average, ≤1.5%; Supplementary Table S3). The low abundance of other phyla (<3% for *Fibrobacteres*, *Spirochaetes*, *Synergistetes*, SR1, *Elusimicrobia*, *Lentisphaerae*, *Candidatus Saccharibacteria*, *Chloroflexi*, *Actinobacteria*, *Cyanobacteria*/*Chloroplast, Verrucomicrobia*, *Armatimonadetes* and *Fusobacteria*) is also consistent with their identification as minor groups in previous analysis of rumen microbiota by NGS techniques^11,28^. The proportion of unclassified sequences at the phylum level was lower in bovine than ovine cultures (on average, 5 vs. 11%, respectively), but within the common range for Ion Torrent NGS studies of the rumen bacterial community^19,23,29^.

The *Bacteroidetes* phylum contained a high percentage of sequences that could not be classified at lower taxonomic levels (66.6% on average; Table 1 and Supplementary Table S2). Previous studies reported a variable proportion of unclassified sequences within this phylum and, although some authors assigned most of them to lower taxonomic levels^10,11,30^, other articles described high proportions of unclassified OTU within this phylum (up to approx. 50%^23,31,32^). In the present assay, the most abundant family was *Prevotellaceae* (10–17% of total sequences), which was negatively affected by EPA and DHA in both ruminant species (*P* < 0.01). *Prevotellaceae* is generally the major bacterial family in the digestive content of ruminants^11,31^. The proportion of *Prevotella*, the most abundant genus among those classified (4–11%), decreased with the three PUFA (*P* < 0.01), being reduced to approximately half by DHA. These results contrast with the resistance of pure cultures of several *Prevotella* strains to EPA and DHA addition^7^, as well as with the lack of effects of marine lipids in some *in vivo* assays in cattle and sheep^11,33^. This inconsistency may be explained by the higher level of PUFA supplementation in the present assay. In any event, *Prevotella* is a very heterogeneous group of bacteria^34^ and, although their best-known function is carbohydrate degradation^35^, some strains have been suggested to play a role in ruminal C18 BH, specifically in 18:0 formation^8^. In our trial, both *Prevotella* (Table 1) and 18:0 concentration in digesta^6^ were reduced in response to PUFA treatments (*r* = 0.915 and *r* = 0.906 for correlations between both variables in cows and sheep, respectively; *P* < 0.001, Supplementary Fig. S3). Within the *Prevotellaceae* family, EPA and DHA caused comparable increases in the abundance of *Hallella* in cattle and *Paraprevotella* in sheep (interaction Sp × PUFA, *P* < 0.05, Fig. 2), which might indicate that perhaps the two genera occupy a similar ecological niche and perform close metabolic functions in each ruminant species^35^. In this regard, *trans* 18:1 concentration was positively correlated with *Hallella* in bovine and ovine cultures (*r* = 0.820 and *r* = 0.805, respectively; *P* < 0.01) and with *Paraprevotella* only in sheep (*r* = 0.732; *P* = 0.007, Supplementary Fig. S3). Altogether, changes in the *Prevotellaceae* family, including those in unclassified sequences (*P* < 0.05), would support the previously suggested hypothesis of a putative role in BH^7,11,33^.

Also in the *Bacteroidetes* phylum, the *Porphyromonadaceae* family was favoured by the inclusion of EPA and DHA (*P* < 0.05; Table 1). This response was partly due to changes in *Barnesiella*, which was not only more abundant in cattle but also showed a stronger response than in sheep (interaction Sp × PUFA, *P* < 0.001; Fig. 2). On the contrary, DPA did not affect *Barnesiella* abundance in ewes and induced lower increases than EPA and DHA in cows. In general, variations in this genus were similar to those observed in total *trans* 18:1 in the companion study^6^ (*r* = 0.961 and *r* = 0.958 in cows and sheep, respectively; *P* < 0.001, Supplementary Fig. S3), and in the rumen bacterial community of cattle receiving n-3 PUFA-rich plant oils^13,36^, which might support a candidate role in ruminal lipid metabolism. *Tannerella*, which is mostly found in the rumen fluid fraction^37^, was the sole genus of the *Porphyromonadaceae* family that seemed to be negatively affected by the n-3 PUFA, although only in cows (interaction Sp × PUFA, *P* < 0.05; Fig. 2). Likewise, increases in *Phocaeicola* abundance (*Bacterioidales inc. sed*. family) were only found in bovine cultures with EPA and DHA (interaction Sp × PUFA, *P* < 0.01; Fig. 2).

Compared with *Bacteroidetes*, the *Firmicutes* phylum contained a greater proportion of classified sequences at the family level (>50%; Table 1 and Supplementary Table S2). The most abundant was *Ruminococcaceae* (6.7 and 4.2% of total sequences in cattle and sheep, respectively), which was impaired by DHA (*P* < 0.01). Within this family, approximately 90% of the sequences could not be classified at lower levels and the genus *Ruminococcus* was always a minor component, even though its abundance in bovine cultures increased by 73% with DHA and 124% with EPA (interaction Sp × PUFA, *P* < 0.01; Fig. 2). Based on their response to marine lipid supplements, *Ruminococcus* and other *Ruminococcaceae* groups have been related to *in vivo* C18 BH in cattle and sheep^8,11^. In the present trial, the abundance of *Oscillibacter*, another genus of this family, was reduced with EPA and DHA, but only in ewes, whereas it remained very low and stable in cows (interaction Sp × PUFA, *P* < 0.01; Fig. 2).

The *Lachnospiraceae* family comprises the main genera with confirmed biohydrogenating ability (i.e., *Butyrivibrio* and *Pseudobutyribibrio*^38,39^), including *B. proteoclasticus* P-18, the only strain demonstrated to metabolise DHA so far^40^. In pure cultures, PUFA addition was shown to seriously affect the growth of this bacterial group^41^. However, marine lipid-induced changes in their *in vivo* abundance were hardly detected or limited to some sub-groups^3,4,9^. Similar responses have also been observed in mixed cultures of ruminal microorganisms^27^. In our work, the significant reductions in the low abundance of *Butyrivibrio* after PUFA addition (Table 1 and Supplementary Table S2) were accompanied by similar decreases in *Pseudobutyrivibrio* (*P* < 0.10), which might have some relationship with decreases in 18:0 concentration in digesta^6^. However, although *Butyrivibrio* and 18:0 proportions showed a significant correlation in bovine (*r* = 0.745, *P* = 0.005) and a tendency in ovine (*r* = 0.568, *P* = 0.054), *Pseudobutyrivibrio* was only marginally correlated with the final product of C18 BH in cows (*r* = 0.502, *P* = 0.096) and not in sheep (*r* = 0.036, *P* = 0.91, Supplementary Fig. S4). In this regard, most *in vivo* studies have failed to find an association between the abundance of these bacterial groups and rumen BH metabolites^8,14,42^, although the functional activity of microbes may not necessarily be proportional to the abundance of their 16 S rRNA gene^43^. Information derived from pure culture tests would help characterizing the metabolic activity of candidate biohydrogenating bacteria, but strain-specific behavior and current technical limitations (e.g., description of specific growth conditions, simulation of the rumen environment, etc.) represent important challenges.

Minor families of the same phylum (Table 1 and Supplementary Table S2), such as *Veillonellaceae* (0.3–0.6%) or *Acidaminococcaceae* (0.5–0.8%), increased their percentage with PUFA addition exclusively in bovine cultures, mainly due to changes in *Megasphaera* and *Succiniclasticum* genera (interaction Sp × PUFA, *P* < 0.01; Fig. 2). In this regard, although the suggested ability of *Megasphaera elsdenii* to produce *trans*-10 *cis*-12 CLA remains controversial^44–46^, the results of our trial seem to support it, according to the correlation analysis between their ruminal proportions in cows (*r* = 0.715, *P* = 0.009) and, to a lower extent, in sheep (*r* = 0.572, *P* = 0.052, Supplementary Fig. S4). Within the *Veillonellaceae* family, the species *Quinella ovalis* has been proposed to have a candidate role in the hydration of unsaturated FA to 10-oxo-18:0, a metabolic pathway favoured by fish oil supplementation^14,25^. Although the databases did not allow identifying *Quinella* in the present trial, unclassified *Veillonellaceae* sequences seemed to increase their percentage in a similar manner to 10-oxo-18:0 in bovine cultures^6^ (*r* = 0.598; *P* = 0.040).

Regarding *Succiniclasticum*, its relative abundance was greater in DPA, EPA and DHA treatments in cattle, whereas numerical increases in sheep were only significant with DHA (*P* < 0.05 for the interaction; Fig. 2). Furthermore, this bacterium was correlated with propionate concentration in cows (*r* = 0.892; *P* < 0.001), but not in ewes (*r* = 0.282; *P* = 0.38, Supplementary Fig. S4). Given that *Succiniclasticum* uses succinate to produce propionate^35,47^, these results might be related to the similar PUFA-induced changes in the percentage of propionate in bovine cultures and the abundance of *Barnesiella* or *Hallela* (*r* = 0.907 and *r* = 0.812, respectively; *P* < 0.001, Supplementary Fig. S3), two succinate-producing bacteria. In ovine, low to moderate correlations were found for the latter genus and propionate (*r* = 0.527; *P* = 0.079) or this VFA and the succinate producer *Paraprevotella* (*r* = 0.647; *P* = 0.023). A putative association between propionate formation and the shift in C18 BH pathways due to PUFA treatment was previously speculated in Toral *et al*.^6^. The analysis presented here would suggest that this relationship is accompanied by increases in populations related to succinate metabolism, which might also play a role in BH. In this regard, a strong relationship was observed between *Barnesiella* and proportions of 18:0 (*r* = −0.861 and *r* = −0.915 in cows and sheep, respectively; *P* < 0.001) or *trans*-11 18:1 (*r* = 0.953 and *r* = 0.917 in cows and sheep, respectively; *P* < 0.001, Supplementary Fig. S3). Similar correlations were observed for *Succiniclasticum* (Supplementary Fig. S4). Another genus of the *Firmicutes* phylum, *Anaerovorax*, has previously been suggested as a candidate biohydrogenating bacterium^8^. In the present study, *Anaerovorax* was favoured by EPA and DHA in both ruminant species (*P* < 0.01; Table 1), but correlations with 18:0 and *trans*-11 18:1 were only significant in cows (*r* = −0.833 and *r* = 0.859, respectively; *P* < 0.001).

The *Proteobacteria* phylum was more abundant in bovine than in ovine cultures (10.4 vs. 4.3%, respectively; Fig. 2) and was affected by PUFA treatment only in cattle (interaction Sp × PUFA, *P* < 0.001). Within this phylum, EPA significantly increased the concentration of *Succinivibrio* and, particularly, of *Ruminobacter* in cows (*P* < 0.05 for the interaction; Fig. 2). In 1970, Yokoyama and Davis^48^ suggested a possible biohydrogenating activity of *Succinivibrio dextrinosolvens*, and more recently, a similar role was tentatively proposed for *Ruminobacter*^26^, which would merit further research.

*Anaeroplasma* (2–8% of total sequences) represented the most abundant genus in the *Tenericutes* phylum, and was negatively affected by EPA and, especially, DHA addition in both species (*P* < 0.001; Table 1). However, we are not aware of other studies that suggest a tentative involvement of *Anaeroplasma* in rumen lipid metabolism.

Finally, the proportion of the minor *Fibrobacteres* phylum increased with the n-3 PUFA treatments in both cattle and sheep (*P* < 0.01; Table 1), whereas increments in *Spirochaetes* and *Synergistetes* phyla abundances were only detected in bovine cultures, in line with variations in their respective major genera, *Sphaerochaeta* and *Jonquetella* (interaction Sp × PUFA, *P* < 0.05; Fig. 2). As discussed previously, additional research would be needed to provide insight into the metabolic pathways of these microbial groups and other candidate biohydrogenating bacteria.

## Conclusions

Both EPA and DHA altered the bacterial community of *in vitro* batch cultures inoculated with rumen microorganisms from cattle and sheep, whereas, consistent with the first of the two initial hypotheses, DPA had a lower effect in bovine and, particularly, in ovine rumen fluid. The three very long chain n-3 PUFA caused changes in the relative abundance of phyla, families and genera of bacteria, such as *Prevotella*, *Barnesiella*, *Ruminococcus*, *Butyrivibrio*, *Anaerovorax*, *Succinivibrio* or *Ruminobacter*, which might suggest a potential association with ruminal C18 BH. Although general responses to PUFA treatments were comparable in cattle and sheep, which would challenge our second initial hypothesis, there were also important variations exclusive to each ruminant species. For example, the abundance of *Hallella*, *Tannerella*, *Phocaeicola*, *Ruminococcus*, *Megasphaera* or *Ruminobacter* was only modified in bovine cultures, whilst *Paraprevotella* and *Oscillibacter* were only affected in ovine. Further research is necessary to examine the metabolic activity of these bacteria and determine if they are truly involved in BH in cattle and sheep.

## Supplementary information

Supplementary Material
