# Supplementary material for: Rumen bacterial community responses to DPA, EPA and DHA in cattle and sheep: A comparative in vitro study
Source: Sci Rep. 2019 Aug 14;9:11857. doi: 10.1038/s41598-019-48294-y (PMC6694141; doi:10.1038/s41598-019-48294-y)
Supplement: Supplementary file 1 — Supplementary Material [file 41598_2019_48294_MOESM1_ESM.docx]

SUPPLEMENTARY MATERIAL

**Rumen bacterial community responses to DPA, EPA and DHA in cattle and sheep**

D. Carreño^1,2^, P. G. Toral^1^*, E. Pinloche^2^, A. Belenguer^1^, D. R. Yáñez-Ruiz^3^, G. Hervás^1^, N. R. McEwan^2,4^, C. J. Newbold^2,5^ and P. Frutos^1^

^1^Instituto de Ganadería de Montaña (CSIC-Universidad de León), Finca Marzanas s/n, 24346 Grulleros, León, Spain

^2^Institute of Biological, Environmental and Rural Sciences (IBERS), Animal and Microbial Sciences, Aberystwyth University, Aberystwyth, Ceredigion, SY23 3EB, United Kingdom

^3^Estación Experimental del Zaidín (CSIC), Profesor Albareda 1, 18008 Granada, Spain

^4^School of Pharmacy and Life Sciences, Robert Gordon University, Aberdeen, AB10 7GJ, United Kingdom

^5^Scotland’s Rural College (SRUC), Kings Buildings, Edinburgh, EH9 3JG, United Kingdom

^*^Corresponding author: pablo.toral@csic.es

**Supplementary Table S1.** Multivariate analysis of variance (MANOVA) conducted with relative abundances of operational taxonomic units (OTU) detected after 24 h of in vitro incubation with rumen inocula of cattle and sheep^1^.

|  | *P*-value^2^ | |
| --- | --- | --- |
| Pairwise comparison | Sheep | Cattle |
| Control vs. DPA | 0.232 | 0.030 |
| Control vs. EPA | 0.020 | 0.024 |
| Control vs. DHA | 0.012 | 0.012 |
| DPA vs. EPA | 0.048 | 0.014 |
| DPA vs. DHA | 0.014 | 0.016 |
| EPA vs. DHA | 0.367 | 0.333 |

^1^The incubated substrate was a total mixed ration containing no additional PUFA (control) or supplemented with 2% dry matter of docosapentaenoic acid (DPA), eicosapentaenoic acid (EPA), or docosahexaenoic acid (DHA).

^2^Adjusted for multiple comparisons using Benjamini and Hochberg’s method. The *P*-values for the fixed effects were as follows: ruminant species, 0.001; PUFA treatment, 0.096; and their interaction, 0.559.

**Supplementary Table S2.** Relative frequencies (% of total sequences, non-transformed values) of bacterial 16S rRNA gene sequences of relevant phyla, families and genera after 24 h of in vitro incubation with rumen inocula of cattle and sheep^1^. Results from the statistical analysis (log-transformed values) are reported in Table 1.

|  | Species | | PUFA treatment | | | |
| --- | --- | --- | --- | --- | --- | --- |
| Taxonomic identification | Cattle | Sheep | Control | DPA | EPA | DHA |
| *Bacteroidetes* | 52.3 | 56.7 | 55.6 | 55.8 | 51.2 | 55.6 |
| Unclassified | 35.0 | 37.8 | 35.2 | 37.2 | 33.9 | 39.4 |
| *Prevotellaceae* | 14.2 | 13.1 | 16.9 | 14.7 | 12.2 | 10.8 |
| Unclassified | 5.28 | 4.31 | 5.13 | 5.10 | 4.65 | 4.21 |
| *Prevotella* | 7.02 | 6.85 | 10.29 | 7.66 | 5.32 | 4.48 |
| *Hallella* | 0.40 | 1.77 | 0.94 | 1.05 | 1.17 | 1.19 |
| *Paraprevotella* | 1.50 | 0.13 | 0.52 | 0.78 | 1.05 | 0.90 |
| *Porphyromonadaceae* | 2.06 | 5.22 | 2.78 | 3.13 | 4.24 | 4.42 |
| Unclassified | 0.34 | 4.58 | 2.08 | 2.18 | 2.76 | 2.81 |
| *Barnesiella* | 1.69 | 0.57 | 0.63 | 0.89 | 1.43 | 1.57 |
| *Tannerella* | 0.03 | 0.08 | 0.08 | 0.05 | 0.05 | 0.04 |
| *Bacteroidales inc. sed.* | 0.89 | 0.27 | 0.42 | 0.53 | 0.64 | 0.73 |
| *Phocaeicola* | 0.89 | 0.27 | 0.42 | 0.53 | 0.64 | 0.73 |
| *Firmicutes* | 19.9 | 16.4 | 17.9 | 17.7 | 18.9 | 18.1 |
| Unclassified | 7.73 | 8.03 | 7.34 | 7.69 | 8.35 | 8.15 |
| *Ruminococcaceae* | 6.72 | 4.18 | 5.84 | 5.38 | 5.49 | 5.09 |
| Unclassified | 6.24 | 3.54 | 5.32 | 4.84 | 4.88 | 4.53 |
| *Ruminococcus* | 0.19 | 0.09 | 0.11 | 0.11 | 0.19 | 0.16 |
| *Oscillibacter* | 0.04 | 0.33 | 0.20 | 0.20 | 0.18 | 0.16 |
| *Lachnospiraceae* | 3.64 | 2.01 | 3.19 | 2.69 | 2.79 | 2.62 |
| Unclassified | 1.73 | 1.33 | 1.55 | 1.54 | 1.57 | 1.46 |
| *Butyrivibrio* | 1.32 | 0.30 | 1.07 | 0.71 | 0.74 | 0.71 |
| *Pseudobutyrivibrio* | 0.36 | 0.30 | 0.40 | 0.30 | 0.32 | 0.30 |
| *Roseburia* | 0.03 | 0.01 | 0.01 | 0.01 | 0.03 | 0.02 |
| *Veillonellaceae* | 0.35 | 0.54 | 0.35 | 0.45 | 0.53 | 0.44 |
| Unclassified | 0.05 | 0.51 | 0.25 | 0.33 | 0.30 | 0.25 |
| *Megasphaera* | 0.11 | 0.00 | 0.01 | 0.03 | 0.11 | 0.07 |
| *Selenomonas* | 0.05 | 0.01 | 0.02 | 0.03 | 0.04 | 0.03 |
| *Anaerovibrio* | 0.08 | 0.02 | 0.04 | 0.05 | 0.06 | 0.05 |
| *Acidaminococcaceae* | 0.57 | 0.72 | 0.50 | 0.61 | 0.72 | 0.76 |
| *Succiniclasticum* | 0.57 | 0.72 | 0.49 | 0.61 | 0.72 | 0.76 |
| *Clostridiales inc. sed. XIII* | 0.64 | 0.41 | 0.39 | 0.48 | 0.64 | 0.59 |
| *Anaerovorax* | 0.46 | 0.27 | 0.29 | 0.32 | 0.44 | 0.41 |
| *Proteobacteria* | 10.4 | 4.27 | 5.66 | 5.82 | 10.2 | 7.56 |
| Unclassified | 5.17 | 1.68 | 3.04 | 3.15 | 4.06 | 3.45 |
| *Succinivibrionaceae* | 4.77 | 2.31 | 2.20 | 2.14 | 5.94 | 3.89 |
| *Succinivibrio* | 0.37 | 0.04 | 0.12 | 0.16 | 0.32 | 0.23 |
| *Ruminobacter* | 4.29 | 1.82 | 1.85 | 1.73 | 5.28 | 3.36 |
| *Tenericutes*^2^ | 4.56 | 5.43 | 7.81 | 5.31 | 4.46 | 2.38 |
| *Anaeroplasma* | 4.55 | 5.39 | 7.78 | 5.28 | 4.44 | 2.36 |
| *Fibrobacteres*^3^ | 2.03 | 2.24 | 1.15 | 1.79 | 2.63 | 2.97 |
| *Spirochaetes* | 2.00 | 2.18 | 1.78 | 2.02 | 2.19 | 2.38 |
| *Spirochaetaceae* | 1.96 | 2.04 | 1.67 | 1.92 | 2.10 | 2.32 |
| *Sphaerochaeta* | 1.30 | 1.55 | 1.07 | 1.35 | 1.54 | 1.74 |
| *Synergistetes*^4^ | 0.82 | 0.67 | 0.35 | 0.60 | 0.94 | 1.09 |
| *Jonquetella* | 0.80 | 0.63 | 0.34 | 0.58 | 0.91 | 1.04 |
| Other phyla^5^ | 3.04 | 1.24 | 2.36 | 2.21 | 1.84 | 2.15 |

^1^The incubated substrate was a total mixed ration containing no additional PUFA (control) or supplemented with 2% dry matter of docosapentaenoic acid (DPA), eicosapentaenoic acid (EPA), or docosahexaenoic acid (DHA).

^2^The *Anaeroplasmataceae* family comprises > 99% of sequences within this phylum.

^3^The *Fibrobacter* genus comprises > 99% of sequences within this phylum.

^4^The *Synergistaceae* family comprises > 99% of sequences within this phylum.

^5^Sum of SR1, *Elusimicrobia*, *Lentisphaerae*, *Candidatus Saccharibacteria*, *Chloroflexi*, *Actinobacteria*, *Cyanobacteria/Chloroplast*, *Verrucomicrobia*, *Armatimonadetes* and *Fusobacteria.*

**Supplementary Table S3.** Relative frequencies (% of total sequences, non-transformed values) of bacterial 16S rRNA gene sequences of relevant phyla, families and genera in the rumen inocula of cattle and sheep (n = 3).

|  | Cattle | | Sheep | |
| --- | --- | --- | --- | --- |
| Taxonomic identification | Mean | SEM^1^ | Mean | SEM^1^ |
| *Bacteroidetes* | 59.6 | 3.59 | 61.3 | 0.99 |
| Unclassified | 42.8 | 0.79 | 33.0 | 1.50 |
| *Prevotellaceae* | 14.8 | 2.86 | 21.2 | 1.21 |
| Unclassified | 6.47 | 0.409 | 6.22 | 0.243 |
| *Prevotella* | 6.87 | 2.509 | 13.1 | 1.23 |
| *Hallella* | 1.08 | 0.034 | 0.11 | 0.022 |
| *Paraprevotella* | 0.34 | 0.058 | 1.73 | 0.170 |
| *Porphyromonadaceae* | 1.25 | 0.035 | 6.27 | 0.276 |
| Unclassified | 0.17 | 0.014 | 5.67 | 0.286 |
| *Barnesiella* | 1.05 | 0.058 | 0.52 | 0.076 |
| *Tannerella* | 0.03 | 0.025 | 0.06 | 0.012 |
| *Bacteroidales inc. sed.* | 0.48 | 0.099 | 0.30 | 0.029 |
| *Phocaeicola* | 0.48 | 0.099 | 0.30 | 0.029 |
| *Firmicutes* | 25.3 | 1.57 | 17.3 | 0.75 |
| Unclassified | 9.44 | 1.185 | 7.69 | 0.390 |
| *Ruminococcaceae* | 11.4 | 0.21 | 5.33 | 0.427 |
| Unclassified | 10.7 | 0.16 | 4.28 | 0.366 |
| *Ruminococcus* | 0.30 | 0.042 | 0.17 | 0.016 |
| *Oscillibacter* | 0.06 | 0.009 | 0.56 | 0.047 |
| *Lachnospiraceae* | 2.56 | 0.148 | 1.70 | 0.170 |
| Unclassified | 1.34 | 0.066 | 1.25 | 0.096 |
| *Butyrivibrio* | 0.79 | 0.087 | 0.17 | 0.046 |
| *Pseudobutyrivibrio* | 0.23 | 0.046 | 0.19 | 0.030 |
| *Roseburia* | 0.02 | 0.017 | 0.01 | 0.000 |
| *Veillonellaceae* | 0.67 | 0.271 | 0.72 | 0.066 |
| Unclassified | 0.08 | 0.028 | 0.65 | 0.074 |
| *Megasphaera* | 0.06 | 0.019 | - | - |
| *Selenomonas* | 0.21 | 0.115 | 0.04 | 0.012 |
| *Anaerovibrio* | 0.24 | 0.129 | 0.03 | 0.005 |
| *Acidaminococcaceae* | 0.30 | 0.114 | 0.81 | 0.078 |
| *Succiniclasticum* | 0.30 | 0.114 | 0.80 | 0.081 |
| *Clostridiales inc. sed. XIII* | 0.69 | 0.047 | 0.47 | 0.064 |
| *Anaerovorax* | 0.36 | 0.039 | 0.28 | 0.033 |
| *Proteobacteria* | 6.69 | 1.649 | 4.93 | 0.517 |
| Unclassified | 5.27 | 1.394 | 3.07 | 0.168 |
| *Succinivibrionaceae* | 0.78 | 0.130 | 1.48 | 0.353 |
| *Succinivibrio* | 0.33 | 0.060 | 0.37 | 0.361 |
| *Ruminobacter* | 0.41 | 0.183 | 0.44 | 0.299 |
| *Tenericutes*^2^ | 0.56 | 0.120 | 1.51 | 0.620 |
| *Anaeroplasma* | 0.55 | 0.118 | 1.47 | 0.614 |
| *Fibrobacteres*^3^ | 0.82 | 0.111 | 0.98 | 0.108 |
| *Spirochaetes* | 0.53 | 0.067 | 0.98 | 0.197 |
| *Spirochaetaceae* | 0.50 | 0.068 | 0.94 | 0.190 |
| *Sphaerochaeta* | 0.39 | 0.063 | 0.80 | 0.174 |
| *Synergistetes*^4^ | 0.12 | 0.003 | 0.30 | 0.048 |
| *Jonquetella* | 0.12 | 0.003 | 0.29 | 0.042 |
| Other phyla^5^ | 2.74 | 0.682 | 1.10 | 0.217 |

^1^SEM = standard error of the mean.

^2^The *Anaeroplasmataceae* family comprises > 97% of sequences within this phylum.

^3^The *Fibrobacter* genus comprises 100% of sequences within this phylum.

^4^The *Synergistaceae* family comprises 100% of sequences within this phylum.

^5^Sum of SR1, *Elusimicrobia*, *Lentisphaerae*, *Candidatus Saccharibacteria*, *Chloroflexi*, *Actinobacteria*, *Cyanobacteria/Chloroplast*, *Verrucomicrobia*, *Armatimonadetes* and *Fusobacteria.*

**Supplementary Figure S1.** Cluster analysis of sequencing profiles based on the method of maximum distance or minimum similarity (complete-linkage) and the Bray-Curtis distances, of bacterial 16S rRNA gene sequences after 24 h of in vitro incubation with rumen inocula of cattle and sheep. The incubated substrate was a total mixed ration containing no additional PUFA (control; black) or supplemented with 2% dry matter of docosapentaenoic acid (DPA; orange), eicosapentaenoic acid (EPA; blue), or docosahexaenoic acid (DHA; green). Values in parenthesis indicate the statistical replicate.

**Supplementary Figure S2.** Relative abundances of bacterial phyla (% of total sequences, non-transformed values) after 24 h of in vitro incubation with rumen inocula of cattle and sheep. The incubated substrate was a total mixed ration containing no additional PUFA (control) or supplemented with 2% dry matter of docosapentaenoic acid (DPA), eicosapentaenoic acid (EPA), or docosahexaenoic acid (DHA). The group "other phyla" includes SR1, *Elusimicrobia*, *Lentisphaerae*, *Candidatus Saccharibacteria*, *Actinobacteria*, *Cyanobacteria/Chloroplast*, *Chloroflexi*, *Verrucomicrobia*, *Armatimonadetes* and *Fusobacteria*.

**Supplementary Figure S3.** Relationships between relative abundances (log-transformed data) of relevant genera of *Bacteroidetes* phylum and ruminal C18 FA concentrations and fermentation parameters, after 24 h of in vitro incubation with rumen inocula of cattle and sheep. The incubated substrate was a total mixed ration containing no additional PUFA (control) or supplemented with 2% dry matter of docosapentaenoic acid (DPA), eicosapentaenoic acid (EPA), or docosahexaenoic acid (DHA). Pearson correlation coefficients (*r*) and *P*-values are reported in each panel.

**Supplementary Figure S4.** Relationships between relative abundances (log-transformed data) of relevant genera of the *Firmicutes* phylum and ruminal C18 FA concentrations and fermentation parameters, after 24 h of in vitro incubation with rumen inocula of cattle and sheep. The incubated substrate was a total mixed ration containing no additional PUFA (control) or supplemented with 2% dry matter of docosapentaenoic acid (DPA), eicosapentaenoic acid (EPA), or docosahexaenoic acid (DHA). Pearson correlation coefficients (*r*) and *P*-values are reported in each panel.
